# Outcomes of AIDS-associated Kaposi sarcoma in Mozambique after treatment with pegylated liposomal doxorubicin

**DOI:** 10.1186/s13027-020-00341-4

**Published:** 2021-01-07

**Authors:** Matthew E. Coldiron, Ana Gabriela Gutierrez Zamudio, Rolanda Manuel, Gilda Luciano, Barbara Rusch, Iza Ciglenecki, Alex Telnov, Rebecca F. Grais, Laurence Toutous Trellu, Lucas Molfino

**Affiliations:** 1 Epicentre, 14-34 Avenue Jean Jaurès, 75019 Paris, France; 2 Médecins Sans Frontières, Maputo, Mozambique; 3 Ministry of Health, Maputo, Mozambique; 4 Médecins Sans Frontières, Geneva, Switzerland; 5 University Hospitals of Geneva, Geneva, Switzerland

**Keywords:** Kaposi sarcoma, Acquired immunodeficiency syndrome, Mozambique, AIDS-related opportunistic infections, Doxorubicin

## Abstract

**Background:**

Kaposi’s sarcoma (KS) is a common HIV-associated malignancy frequently associated with poor outcomes. It is the most frequently diagnosed cancer in major cities of Mozambique. Antiretroviral therapy is the cornerstone of KS treatment, but many patients require cytotoxic chemotherapy. The traditional regimen in Mozambique includes conventional doxorubicin, bleomycin and vincristine, which is poorly tolerated. In 2016, pegylated liposomal doxorubicin was introduced at a specialized outpatient center in Maputo, Mozambique.

**Methods:**

We performed a prospective, single-arm, open-label observational study to demonstrate the feasibility, safety, and outcomes of treatment with pegylated liposomal doxorubicin (PLD) in patients with AIDS-associated Kaposi sarcoma (KS) in a low-resource setting. Chemotherapy-naïve adults with AIDS-associated KS (T1 or T0 not responding to 6 months of antiretroviral therapy) were eligible if they were willing to follow up for 2 years. Patients with Karnofsky scores < 50 or contraindications to PLD were excluded. One hundred eighty-three patients were screened and 116 participants were enrolled. Patients received PLD on three-week cycles until meeting clinical stopping criteria. Follow-up visits monitored HIV status, KS disease, side effects of chemotherapy, mental health (PHQ-9) and quality of life (SF-12). Primary outcome measures included vital status and disease status at 6, 12, and 24 months after enrollment.

**Results:**

At 24 months, 23 participants (20%) had died and 15 (13%) were lost to follow-up. Baseline CD4 < 100 was associated with death (HR 2.7, 95%CI [1.2–6.2], *p* = 0.016), as was T1S1 disease compared to T1S0 disease (HR 2.7, 95%CI [1.1–6.4], *p* = 0.023). Ninety-two participants achieved complete or partial remission at any point (overall response rate 80%), including 15 (13%) who achieved complete remission. PLD was well-tolerated, and the most common AEs were neutropenia and anemia. Quality of life improved rapidly after beginning PLD.

**Discussion:**

PLD was safe, well-tolerated and effective as first-line treatment of KS in Mozambique. High mortality was likely due to advanced immunosuppression at presentation, underscoring the importance of earlier screening and referral for KS.

## Introduction

Kaposi’s sarcoma (KS) is a major cause of mortality in sub-Saharan Africa, where it is generally associated with HIV [1, 2]. In Mozambique, it is the most frequent cancer documented in cancer registers in the major cities of Maputo and Beira [3], with an estimated age-standardized incidence of 23 cases per 100,000 persons per year and 3215 deaths in 2012 alone [4]. In sub-Saharan Africa, KS can be aggressive and have poor outcomes [5]. KS lesions are often visible and painful, leading to physical and psychological disability, and associated stigma may play a role in delayed presentation [6].

The cornerstone of treatment of HIV-associated KS is antiretroviral therapy (ART), which reduces the burden of KS disease by itself [7, 8]. Nonetheless, with advanced KS, chemotherapy is often necessary [9]. In high-income countries, first-line chemotherapy consists of liposomal anthracyclines or taxanes [10, 11]. Pegylated liposomal formulations of doxorubicin (PLD) are associated with more favorable outcomes and safety profiles than conventional forms [12–15], but are expensive. In low- and middle-income countries, a combination of bleomycin and vincristine (BV), with or without conventional doxorubicin (ABV) is often still the standard of care. These combinations can be poorly-tolerated and associated with lower quality of life, so better treatment options are necessary [16].

In March 2016, Médecins Sans Frontières (MSF) began offering PLD as first-line chemotherapy for KS in Maputo, Mozambique. A prospective observational study was implemented to document safety, tolerability and effectiveness, with an overall goal of improving clinical practice in a low-resource setting.

## Methods

### Study setting

The *Centro de Referencia de Alto-Maé* (CRAM) is a collaboration between MSF and the Mozambican Ministry of Health. This intermediate-level, ambulatory care facility bridges the gap between primary clinics and hospitals by ensuring access to specialized services for HIV-infected patients with complications. Between 2010 and 2015, 1567 HIV-positive patients were treated for KS at CRAM. Most had advanced disease at baseline (62% with T1S0 and 21% with T1S1), received a median of 12 cycles of chemotherapy, and had relatively poor outcomes (36% loss to follow up and 7% died). Over the same period, only 6 HIV-negative KS patients were treated at the CRAM [17].

### Participants

New patients and chemotherapy-naïve established patients were screened for eligibility. Inclusion criteria included the following: age > 15 years; either T1 KS, or else T0 KS not responding to 6 months of ART together with progressive/extensive lesions, B symptoms or significant impact on quality of life as judged by the physician; and willingness to follow up for 2 years. Cutaneous punch biopsy was used to confirm KS, but when not possible (for logistical or staffing reasons), the judgement of two physicians was considered sufficient to establish the diagnosis in the setting of typical advanced stage disease.

Exclusion criteria included history of receipt of doxorubicin (for any reason), Karnofsky score < 50 [18, 19], planned move out of the study area within 2 years, mental impairment leading to inability to contribute to data collection, pregnancy, breastfeeding, and (among women) refusal to use a birth control method other than condoms.

Cardiac function was assessed at screening by history and physical exam; patients with signs or symptoms of congestive heart failure were referred for echocardiography. If cardiac evaluation showed left ventricular ejection fraction > 50% and no structural abnormalities, the patient was eligible.

### Clinical definitions

KS disease was classified according to the ACTG staging system, based on tumor extent (T), immune system status (I), and evidence for HIV-associated systemic illness (S). Each variable is classified as good risk (0) or poor risk (1) [20]. These prognostic factors have been prospectively and independently validated [21].

Clinical outcomes were defined as follows, using previously-described criteria [14]:

- Complete Response (CR): Resolution of any detectable disease for ≥4 weeks including KS associated edema or effusion.
- Partial Response (PR): Absence of new cutaneous or oral lesions, new visceral sites of involvement or the appearance or worsening of tumor-associated edema or effusions. In addition, at least one of the following applied:
  - 50% decrease in the number of previous skin lesions;
  - ≥50% of flattening of all previously raised lesions;
  - ≥50% decrease in the sum of the products of the largest perpendicular diameters of indicator lesions at enrolment;
  - Patient met the criteria for CR except for residual tumor associated edema or effusion;
  - The response lasted ≥4 weeks.
- Overall Response (OR): Partial Response + Complete Response
- Stable Disease: Not meeting the criteria for progression, PR or CR
- Progressive Disease: One or more of the following:
  - New visceral sites of involvement, or progression of visceral disease
  - New or increasing tumor-associated edema or effusion lasting at least 1 week and interfering with normal activities.
  - ≥25% increase in number of skin lesions.
  - A change in the character of ≥25% of previously “flat” skin lesions to “raised”.
  - ≥25% increase in the sum of the products of the largest perpendicular diameters of the indicator skin lesions.

### Dosing of PLD

20 mg/m^2^ of body surface area (BSA) of PLD (Janssen, Belgium) was administered every 3 weeks until CR, or PR after having received at least 4 cycles of PLD, with good reported ART adherence and decreased pain. PLD was stopped after a cumulative dose of 550 mg/m^2^ of PLD.

PLD administration was delayed and rescheduled for the following: < 1000 neutrophils/mm^3^, < 75,000 platelets/mm^3^, and serum creatinine and alanine aminotransferase ≥2 times the upper limit of normal. PLD was held for serum hemoglobin concentrations < 10 g/dl, unless blood transfusion was available immediately following PLD infusion, in which case PLD was administered for hemoglobin between 8 and 10 g/dl.

Patients who previously met criteria for stopping PLD but who had new or worsening lesions, increased pain, or decreased quality of life over the course of follow-up were evaluated for re-initiation.

### Follow-up visits

Study evaluations were scheduled at 3, 6, 9, 12, 18 and 24 months. At these visits, a physician performed a full history and physical exam and measured sentinel KS lesions. Blood samples to monitor for potential PLD toxicities were drawn prior to each PLD infusion, as described above. Blood was also drawn to monitor HIV control at each of the study evaluation visits.

The PHQ-9 questionnaire was administered at enrolment and annually thereafter. This depression screening tool has been validated in at least two sub-Saharan African settings, and scores ≥10 have been shown to be valid for the diagnosis of major depression [22, 23]. Quality of life was assessed using the SF-12® questionnaire, which was administered at enrolment and every 6 months thereafter.

Information regarding the circumstances of death of patients who died during follow-up was conducted by interview with their surviving contacts.

### Safety monitoring

Adverse events (AE) and serious adverse events (SAE) were evaluated at each study visit. An SAE was defined as an AE that resulted in death, was life-threatening, required hospitalization or prolongation of existing hospitalization, resulted in a persistent or significant disability or incapacity, required medical intervention intended to prevent one of the above outcomes, or was judged by the investigator as potentially serious. The causal relationship between PLD and a given AE or SAE was determined following Mozambican guidelines.

### Sample size and statistical analysis

The target sample size was based on overall response. Assuming that 66% of participants would have CR or PR at 12 months [24], we calculated that 86 participants would be needed to show this response rate with a 10% margin. Given an expected transfer rate of 5% and loss to follow up of 30%, a final sample size of 116 participants was set. Baseline characteristics of study participants were described using appropriate measures of frequency, central value and dispersion. Survival was estimated using the Kaplan-Meier method, and Cox-proportional hazard modelling was used to explore associations between baseline characteristics and survival. Participants who achieved PR but later required re-starting of PLD were censored at the time that PLD was restarted. Patients lost to follow-up were censored at the date of their last study visit.

Data was entered using EpiData version 3.0. The scoring of the SF-12® questionnaire was performed using Optum® PRO CoRE Smart Measurement® System, version 1.4.7003.15542. All other analysis was performed using Stata v 15.0.

## Results

### Description of participants

**Fig. 1:**
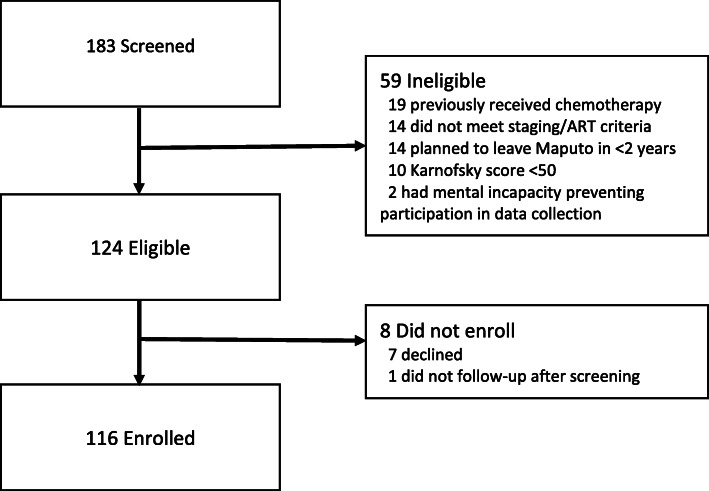
Screening and enrolment

Beginning March 1, 2016, a total of 183 patients were screened and 116 enrolled (Fig. 1). Sociodemographic characteristics of potential participants who were screened but not enrolled were generally similar to enrolled participants (Table 1). Five participants had signs of CHF warranting echocardiography; all had normal ejection fractions and were eventually included. Forty-six percent had been diagnosed with HIV in the 6 months preceding enrolment. Overall, 88% had started (or re-started) ART in the previous 6 months. Participants presented with advanced immunosuppression (median CD4 218, IQR [73–448]), and 93 (80%) were receiving prophylactic co-trimoxazole at the time of enrollment.

**Table 1:** Baseline characteristics of enrolled patients (*N* = 116)

Characteristic	n (%), unless otherwise specified
**Sex**, n(%)	
Male	74 (64)
Female	42 (36)
**Age in years**, n(%)	
15–19	1 (1)
20–29	19 (16)
30–39	55 (47)
40–49	43 (26)
≥ 50	11 (10)
**Educational attainment**, n(%) (*N* = 114)	
No formal education	5 (4)
Primary school	51 (44)
Intermediate school	50 (44)
High school or higher	8 (8)
**Kaposi stage**, n(%)	
T1S0	80 (69)
T1S1	31 (26)
T0S1	2 (2)
T0S0	3 (3)
**Lymphedema present at enrolment**	83 (72)
**Time since HIV diagnosis in months**, n(%)	
0–5	53 (46)
6–11	13 (11)
12–23	18 (16)
≥ 24	32 (28)
**Time on ART in months**, n(%)	
0–5	62 (53)
6–11	13 (11)
12–23	16 (14)
≥ 24	25 (22)
**ART regimen**, n(%)	
Efavirenz-based	107 (92)
Nevirapine-based	3 (3)
Lopinavir/ritonavir or atazanavir-based	6 (5)
**On treatment for TB**, n(%)	19 (16)
**Laboratory values**, median (IQR)	
CD4 (cells/mm3)	218 (73–448)
Serum Hemoglobin (g/dl)	11.0 (9.8–12.4)
Platelets (10^3^ cells/μl)	284 (222–360)
Absolute neutrophil count (10^3^ cells/μl)	2.7 (2.0–3.6)
Creatinine (mg/dl)	0.8 (0.6–1.0)
Alanine aminotransferase (U/L)	11 (6–17)

At enrollment, 115 patients had cutaneous lesions: 40 had lesions too numerous to count, and of the 75 remaining patients, the median number of discrete lesions on the entire body was 17 (IQR: 6–30). In addition, 46% had visible mucosal lesions, and 23% had evidence of lesions in the respiratory or gastrointestinal tracts (by chest radiography, bronchoscopy, or upper endoscopy). All 43 participants (37%) who had biopsies had histologically confirmed KS.

### Outcomes

**Fig. 2:**
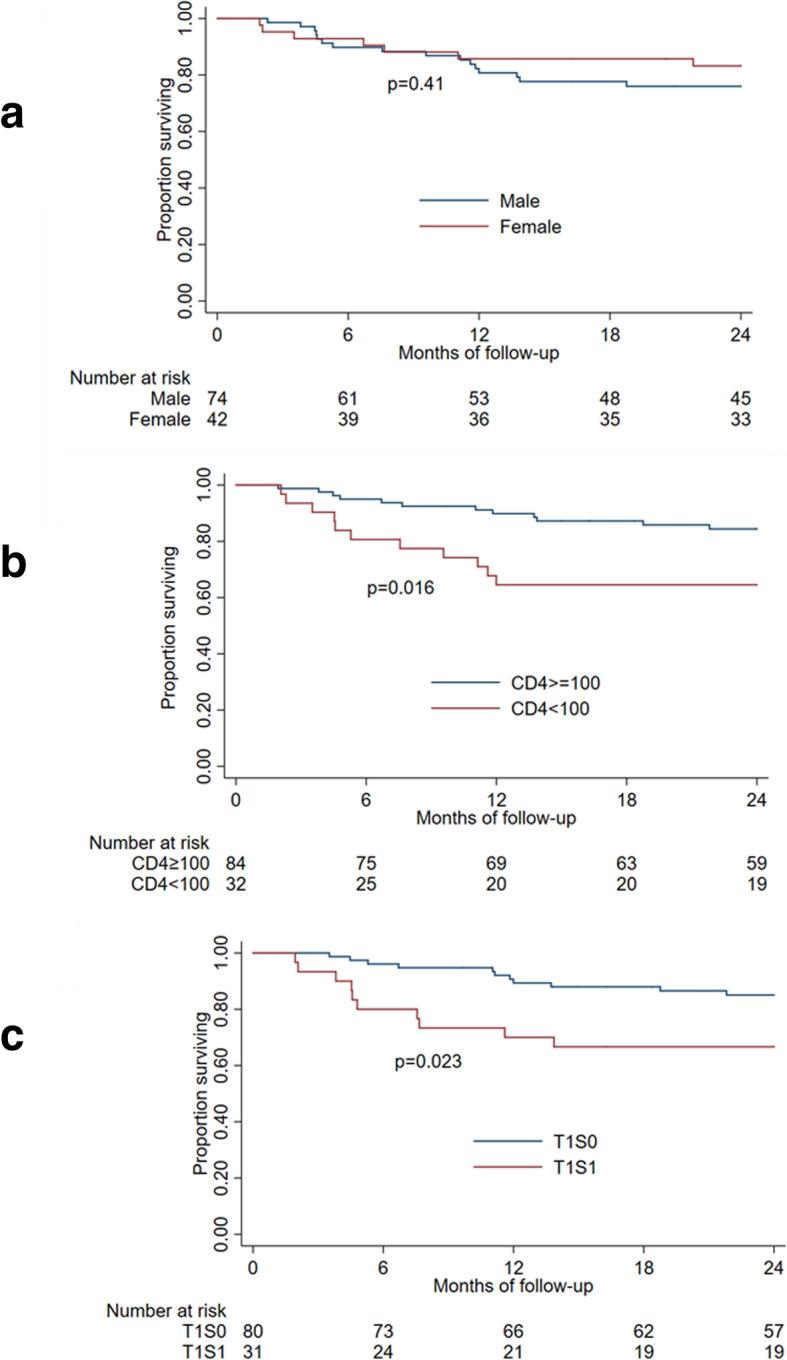
Survival by sex (**a**), baseline CD4 (**b**) and baseline tumor stage (**c**)

At 24 months, 78 patients (67.2%) were known to be alive, 23 (19.8%) had died, and 15 (12.9%) were lost to follow-up. Among the 15 participants lost to follow-up, median time in the study was 335 days (IQR 42–496, range 3–638). In bivariate analysis, there was no difference in survival by sex (*p* = 0.41, Fig. 2a). Participants with CD4 < 100 at enrolment were more likely to die (HR 2.7, 95%CI [1.2–6.2], *p* = 0.016, Fig. 2b), as were participants with T1S1 disease at enrollment compared to those with T1S0 disease (HR 2.7, 95%CI [1.1–6.4], *p* = 0.023, Fig. 2c).

A total of 92 participants achieved CR or PR (OR = 80%), including 15 participants (13%) who achieved complete remission. Among the 92 patients who ever achieved CR or PR, 77 were alive at 24 months of follow-up, 7 were lost to follow-up, and 8 died. Among the 24 patients who never achieved CR or PR, 8 were lost to follow-up and 16 died.

**Fig. 3:**
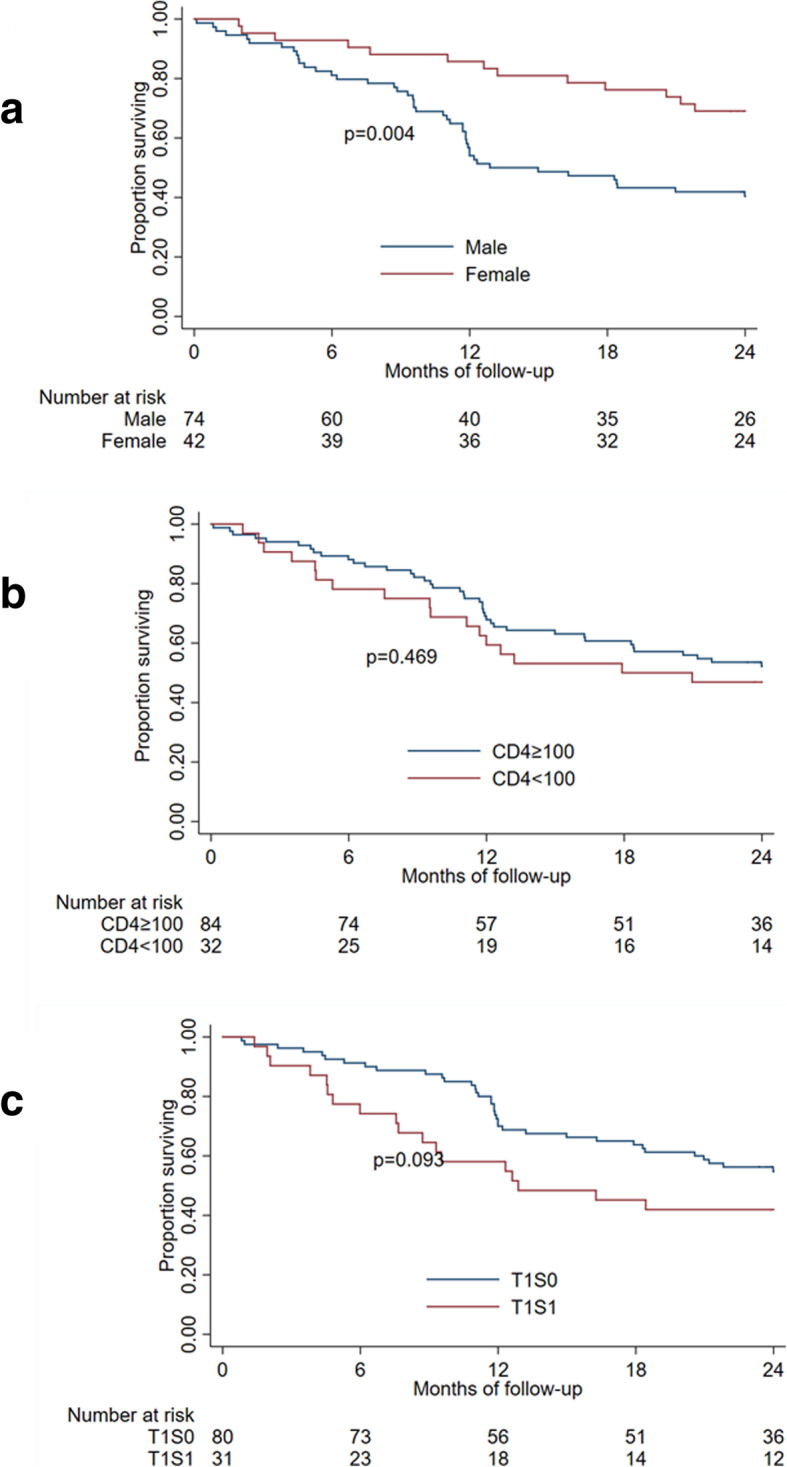
Progression-free survival by sex (**a**), baseline CD4 (**b**) and baseline tumor stage (**c**)

Progression-free survival is presented in Fig. 3. At 1 year, progression-free survival was 65.5% (95%CI [56.1–73.4]), and at 2 years, progression-free survival was 50.7% (95%CI [41.3–59.4]). In contrast to overall survival, male participants were less likely to have progression-free survival at 2 years (HR 0.4, 95%CI [0.2,0.7], *p* = 0.004, Fig. 3a).

Among the 92 participants achieving CR or PR, 74% needed between 5 and 8 cycles of PLD to reach CR or PR for the first time. 26 (28%) of these patients eventually needed to restart PLD due to worsening symptoms or expanding lesion, of whom 20 survived to study exit, 2 were lost to follow up, and 4 died. The median time between achieving CR or PR and restarting PLD was 174 days (IQR 123–316).

### Adverse events

Thirty-four SAEs were notified during the study period, including 23 deaths, 1 immediate transfusion reaction necessitating hospitalization, and 10 hospitalizations.

The circumstances of the 23 deaths were documented, and for many, it was impossible to make a single unifying diagnosis of the cause of death. Social problems (e.g., unstable housing, drug use, family strife) were common co-factors. Fourteen deaths occurred in hospital, and 9 at home. In general terms, they were classified as follows:

- 6 advanced HIV, death directly related to KS
- 6 advanced HIV, death unrelated to KS, but without other confirmed opportunistic infection (OI)
- 4 advanced HIV, with a confirmed other OI that predated study enrolment
- 2 advanced HIV, with a confirmed other OI that developed after study enrolment
- 1 bacterial pneumonia in a patient with CD4 > 500
- 1 likely stroke
- 1 non-neutropenic sepsis of unclear origin
- 1 non-Hodgkin’s lymphoma
- 1 cause of death could not be ascertained

The immediate infusion reaction occurred in a 21-year-old female who developed acute shortness of breath and severe chest pain during her first PLD infusion. She was hospitalized, and symptoms resolved after treatment with hydrocortisone. The participant continued to receive further doses of PLD without incident and achieved partial remission. This was the only direct infusion reaction seen during the 894 doses of PLD administered during the study (0.1%).

The 10 hospitalizations were due to progressive KS (4 patients), TB (2 patients), wasting syndrome (2 patients) and other OIs (2 patients).

Sixty-five AEs were notified in 33 individual participants (28.4% of all enrolled). In terms of severity, 22 (38%) were classified as mild, 31 (53%) as moderate, and 5 (9%) as severe; 46 (73%) were judged linked to PLD. The most common AEs were hematologic abnormalities: neutropenia (12 events), anemia (15 events), neutropenia and anemia (21 events), isolated thrombocytopenia (2 events) and pancytopenia (3 events). Two cases of the hand-foot syndrome were notified. The first occurred in a 42-year old female who had received 6 doses of PLD, and the second occurred in a 36-year old male who had received 2 doses. Both cases had mild palmar and plantar erythema with mild desquamation, and both resolved within 2 weeks without treatment.

### Mental health and quality of life

At enrolment, 10 patients (9%) had PHQ-9 scores ≥10, and of patients with subsequent evaluations, 3 of 102 (3%) had a PHQ-9 score ≥ 10 at their final study visit.

**Fig. 4:**
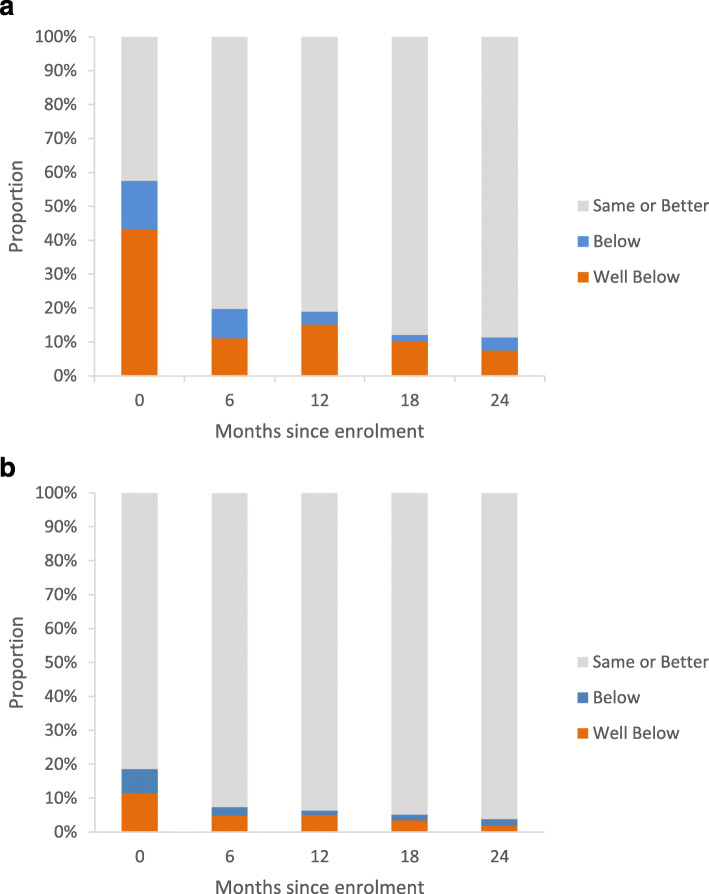
SF-12® scores benchmarked against general population. (**a**) Represents the Physical Component Summary and (**b**) represents the Mental Component Summary

Baseline SF-12® scores were available for 113 participants. At enrolment, 65 patients (58%) had a Physical Component Summary score below that of the general population, including 49 (43%) who scored “Well Below”. This proportion dropped significantly during follow up (Fig. 4a). For the Mental Component Summary, at enrolment, 21 patients (19%) had a score below that of the general population, including 13 (12%) who scored “Well Below”. As with the Physical Component Summary, the proportion of those with scores below that of the general population decreased during follow-up (Fig. 4b). The most marked differences came in the Physical Component Summary in the first 6 months of follow-up. Each of the individual components (Physical functioning, Role physical, Bodily pain, and General health) improved between those two evaluations, and the average score among the cohort for three of the four components went from below the norm for the general population to above the norm.

## Discussion

We describe the outcomes of the first 116 patients to receive PLD in Mozambique. PLD was safe and effective in this setting, and to our knowledge, we have described the mental health and quality of life of patients with HIV-related KS for the first time in Mozambique.

### Effectiveness of PLD

Participants had significant KS disease at baseline, and many also presented with advanced immunosuppression. Despite this, the OR rate was 80%. Given the severity of presentations, it is not surprising that a small minority of patients (13%) ever achieved CR. Importantly, the number of cycles of chemotherapy needed to achieve first response was low (≤8 cycles for 83% of participants achieving CR or PR), much lower than previously reported with BV and ABV regimens in the same clinic, when patients received a median of 12 cycles of chemotherapy [17]. Receiving fewer doses of cytotoxic chemotherapy means fewer side effects, fewer hospitalizations and fewer clinic visits for patients, which is an important benefit of the PLD regimen.

28% of patients who achieved CR or PR eventually needed additional doses of PLD. Although it is impossible to say with certainty, this phenomenon does not seem to be related directly to PLD. A few of these “relapses” occurred after interruption of ART, and others occurred in patients with massive baseline KS burden and significant lymphedema. But a majority occurred in patients with undetectable (or nearly undetectable) HIV viral load. Given that complete immune reconstitution is slow in ideal circumstances, it is unsurprising that some patients needed additional doses of PLD. These findings may also be partially explained by a decreased anti-HHV8 T-cell response that has been described in patients with KS (compared to the anti-HHV8 T-cell response seen in patients with asymptomatic HHV8 infection) [25].

Advanced KS and AIDS at baseline means that CR was likely an impossible goal for many participants. In this setting, it is reasonable for clinicians to focus on alleviation of pain, slowing KS disease progression, and improvement in quality of life, while at the same time achieving virologic suppression. The results suggest that it is reasonable to treat until symptoms have ameliorated (and not until lesions disappear) because many patients with severe baseline disease will eventually need to restart chemotherapy after initial response. Restarting PLD was not necessarily a bad prognostic indicator, as 20 of 26 of our patients who restarted survived until study exit.

### Safety of PLD

Broadly speaking, PLD was safe and well-tolerated. The hematologic toxicities seen were generally of moderate severity, and not unexpected. And although some patients needed transfusions, the side effects of PLD were less limiting and less toxic to the patients than with the previous ABV regimen, and also those described with the use of paclitaxel monotherapy [17, 26]. While data on the incidence of hand-foot syndrome in Sub-Saharan Africa are scarce, it is possible that the relatively lower doses and the three-week interval between doses may explain its rarity in this cohort.

We described an immediate infusion reaction, which was frightening for the patient and for the clinical staff. Acute-onset chest tightness and shortness of breath have been previously described in the literature, particularly during the first infusion of PLD [27]. Importantly, after full work-up, this patient was able to receive further doses of PLD without incident.

### Mortality and loss to follow-up

Overall, mortality was high in this population. At 24 months of follow-up, 20% of study participants had died, and another 13% had been lost to follow-up. Most deaths came within the first year after enrolment, and participants with lower baseline CD4 and more advanced KS disease were more likely to die. When examining the individual causes of death, it becomes clear that most of those who died had complex medical and social situations.

In previous data from the CRAM, few patients receiving chemotherapy (7%) were known to have died, but 36% were lost to follow-up. But a lack of strict contact tracing impeded understanding the reasons for loss to follow-up. Our current results suggest that a significant proportion of patients previously lost to follow-up actually died. Nonetheless, we note that, even in the worst-case scenario, if we assume that all patients lost to follow-up in this study died, the overall mortality in this group of patients receiving PLD (33%) would be less than that of the historical cohort in the CRAM (43%) [17].

It is important to note that these two analyses happened at different points in time. Many of the patients in the historical CRAM cohort were treated in an era when access to ART and VL monitoring were more difficult. There is clear understanding that ART is the cornerstone of KS treatment, and that its addition has greatly improved survival for KS patients [28]. Therefore, the high mortality seen during the first year of treatment underscores the importance of providing a full package of HIV care, including good counseling for ART compliance, VL monitoring, awareness of ART resistance patterns, and access to second- and third-line ART. It is also important to note that the specialized ambulatory clinic where the study took place was in a densely-populated urban setting, where distances and travel time to the clinic generally did not exceed 90 min, which may be different from rural settings.

### Psychosocial well-being

One of the innovations of this study was to objectively monitor the psychosocial well-being of participants. PLD has led to increased quality of life for KS patients in high-income settings [16], but to our knowledge there has been little documentation of this in low-resource settings.

Our most striking finding was the improvement in the physical components of the SF-12® evaluation in the first 6 months of treatment with PLD. At baseline, pain and a sense of general ill-health – both components of the Physical Component Summary – drove quality of life scores to be significantly below those of the general population in many participants. At 6 months, the most important gains in the overall quality of life came because of a reduction in pain. These intuitive results provide evidence of the PLD’s benefits, particularly for advanced lymphedematous presentations commonly seen in this setting, which have a major impact on mobility and morbidity.

### Improving chemotherapy regimens in sub-Saharan Africa

The traditional ABV regimen is not ideal, because of substandard outcomes and an unfavorable side effect profile, so finding less toxic alternatives is urgent. Indeed, paclitaxel was recently shown to be superior to both a combination of bleomycin and vincristine and also to oral etoposide in a multi-centric trial in southern Africa, though we note that paclitaxel toxicities were largely the same compared to the vincristine/bleomycin group [26]. We are unable to compare our observational results with PLD with those from the paclitaxel trial, but future research in Africa should include head-to-head testing of PLD and paclitaxel. These standards of care in high-income settings have only been directly compared in smaller studies, and in Africa should have a special focus on safety and tolerability [29].

Nonetheless, given the lack access to even basic chemotherapy like ABV in most of sub-Saharan Africa [30, 31], this may be difficult. General health systems barriers include lack of medications, lack of clear guidelines, staff with little oncological training, few diagnostic and monitoring tools and poor infrastructure necessary to handle cytotoxic medications [30, 32, 33]. Expanding the use of PLD will therefore require increased supply and additional financing. Since the initiation of this study, PLD now appears on procurement lists of the Global Fund, which is a positive step. But to ensure access to PLD (and paclitaxel), drug manufacturers and international partners will need to work to lower prices, increase production capacity, and improve availability. In the meantime, early diagnosis of HIV and KS should be emphasized, to avoid the late presentations seen commonly in our setting.

### Limitations

Our study has several limitations. First, the observational nature of the study makes it impossible to draw strict comparisons between PLD and previous regimens. Second, the exclusion of non-HIV-associated KS limits our ability to extrapolate on PLD’s effectiveness in other populations. “Endemic” KS is common in Mozambique, and the seroprevalence of HHV8 has been reported as as high as 51% [34]. Unfortunately, this is a population not treated at the specialized study site. Third, lack of specific data regarding ARV adherence limits interpretation of the root cause of relapses, which could be due to poor adherence, or in the setting of good adherence, virological resistance. Finally, the lack of biopsy confirmation in 57% of patients was unavoidable because of logistic and staffing issues.

## Conclusions

These results show the effectiveness, safety, and tolerability of PLD as first-line chemotherapy for KS in a resource-limited setting. Moving from this positive experience in a specialized setting to broader uptake will require efforts to increase access to PLD and other new chemotherapies, integrated into a comprehensive plan to upgrade skills of health staff and infrastructure.

## Data Availability

The datasets used and/or analyses during the current study are available from the corresponding author on reasonable request.

## Abbreviations

ABV: Combination therapy with doxorubicin, bleomycin and vincristine
ACTG: AIDS Clinical Trial Group
AE: Adverse event
AIDS: Acquired immunodeficiency syndrome
ART: Antiretroviral therapy
BSA: Body surface area
BV: Combination therapy with bleomycin and vincristine
CR: Complete remission
CRAM: Centro de Referência de Alto-Maé
HR: Hazard ratio
HIV: Human immunodeficiency virus
IQR: Interquartile range
KS: Kaposi’s sarcoma
MSF: Médecins Sans Frontières
OI: Opportunistic infection
OR: Overall response
PHQ-9: Patient health questionnaire-9
PLD: Pegylated liposomal doxorubicin
PR: Partial remission
SAE: Serious adverse event
